# Competência Prognóstica Distinta entre Modelo Clínico e Anatômico em Síndromes Coronarianas Agudas: Comparação por Tipo de Desfecho

**DOI:** 10.36660/abc.20190062

**Published:** 2020-08-19

**Authors:** Mateus S. Viana, Vitor C. A. Correia, Felipe M. Ferreira, Yasmin F. Lacerda, Gabriela O. Bagano, Leticia L. Fonseca, Lara Q. Kertzman, Milton V. Melo, Marcia M. Noya-Rabelo, Luis C L Correia

**Affiliations:** 1 Escola Bahiana de Medicina e Saúde Pública Salvador BA Brasil Escola Bahiana de Medicina e Saúde Pública,Salvador, BA - Brasil; 2 Hospital São Rafael Salvador BA Brasil Hospital São Rafael,Salvador, BA – Brasil

**Keywords:** Síndrome Coronariana Aguda/fisiopatologia, Aterosclerose, Infarto do Miocárdio, Mortalidade, Doenças Cardiovasculares/prevenção e Controle, Hospitalização, Prognóstico

## Abstract

**Fundamento:**

Eventos isquêmicos recorrentes decorrem de instabilidade de placa aterosclerótica, enquanto morte após um evento isquêmico decorre da gravidade do insulto. A natureza diversa desses tipos de eventos pode fazer com que dados clínicos e anatômicos tenham diferentes capacidades prognósticas a depender do tipo de desfecho.

**Objetivo:**

Identificar as predileções prognósticas de dados clínicos e dados anatômicos em relação a desfechos coronários fatais e não fatais durante hospitalização de pacientes com síndromes coronarianas agudas (SCA).

**Métodos:**

Pacientes consecutivamente admitidos por SCA que realizaram coronariografia foram recrutados. O escore SYNTAX foi utilizado como modelo anatômico e o escore GRACE como modelo clínico. A capacidade preditora desses escores foi comparada quando à predição de desfechos isquêmicos não fatais (infarto ou angina refratária) e de morte cardiovascular durante hospitalização. Significância estatística foi definida por p < 0,05.

**Resultados:**

Entre 365 indivíduos, 4,4% foi a incidência de óbito hospitalar e 11% de desfechos isquêmicos não fatais. Para morte cardiovascular, ambos os escores — SYNTAX e GRACE — apresentaram capacidade discriminatória, com estatísticas-C similares: 0,80 (95%IC: 0,70–0,92) e 0,89 (95%IC 0,81–0,96), respectivamente — p=0,19. Quantos aos desfechos isquêmicos não fatais, o escore SYNTAX apresentou valor preditor (estatística-C = 0,64; 95%IC 0,55–0,73), porém o escore GRACE não mostrou associação com esse tipo de desfecho (estatística-C = 0,50; 95%IC: 0,40–0,61) — p=0,027.

**Conclusão:**

Os modelos clínico e anatômico predizem satisfatoriamente morte cardiovascular em SCA, enquanto a recorrência de instabilidade coronária é melhor prevista por características anatômicas do que por dados clínicos. (Arq Bras Cardiol. 2020; 115(2):219-225)

## Introdução

Modelos multivariados têm sido validados como ferramentas prognósticas em síndromes coronarianas agudas (SCA), constituídos por dados clínicos,^1^ por dados anatômicos^2^ ou pela combinação dos dois.^3^ Esses modelos têm valor preditor reconhecido quanto a eventos recorrentes, porém não está claro se o valor prognóstico varia a depender do tipo de desfecho.

Eventos isquêmicos recorrentes não fatais representam o fenômeno de instabilidade de placa aterosclerótica, enquanto morte após um evento isquêmico decorre da gravidade do insulto e da resistência do organismo. A diferente natureza fisiopatológica desses tipos de eventos pode fazer com que dados clínicos e anatômicos tenham diferentes capacidades prognósticas a depender do tipo de desfecho. Caso isso seja verdade, a generalização do valor prognóstico quanto a “desfechos cardiovasculares” ficaria comprometida, fazendo-se necessária uma individualização da predição de cada modelo para o tipo de desfecho.

Este trabalho tem o objetivo de avaliar e de comparar o valor prognóstico de dados clínicos e anatômicos em relação a desfechos fatais e não fatais em pacientes com SCA. Dessa forma, foi utilizada uma coorte hospitalar de pacientes admitidos nessas condições, sendo o escore GRACE escolhido como representante da predição para dados clínicos e o escore SYNTAX utilizado como preditor com base na anatomia.

## Metodologia

### Seleção da População

Foram selecionados indivíduos consecutivamente admitidos na Unidade Cardiovascular Intensiva de hospital terciário entre julho de 2007 a setembro de 2014, com diagnóstico de SCA. O critério de inclusão foi definido por desconforto precordial típico ou equivalente e em repouso nas últimas 48 horas, associado a pelo menos uma das seguintes características: 1) marcador de necrose miocárdica positivo, definido por troponina T ≥ 0,01 μg/L ou troponina I ≥ 0,034 μg/L, o que correspondem a valores acima do percentil 99;^7 , 8^ 2) alterações eletrocardiográficas isquêmicas, consistindo em inversão de onda T (≥ 0,1 mV) ou desvio do segmento ST (≥ 0,05 mV); 3) doença arterial coronariana documentada, definida por história de infarto do miocárdio ou angiografia prévia demonstrando obstrução coronariana ≥ 50% do diâmetro luminal. Além disso, para inclusão na análise, os pacientes precisavam ter sido submetidos a procedimento de coronariografia durante o internamento. Foram excluídos aqueles que discordaram em participar do registro e/ou submetidos previamente a procedimento de revascularização cirúrgica do miocárdio. O protocolo está em conformidade com a Declaração de Helsinki e foi liberado pelos Comitês de Ética em Pesquisa das Instituições. Todos os pacientes assinaram termo de consentimento livre e esclarecido.

### Escores Preditores (SYNTAX e GRACE)

O cálculo do escore SYNTAX foi feito por um cardiologista intervencionista experiente e cego para o quadro clínico e para os desfechos. Este médico avaliou cada obstrução da árvore coronariana com percentual de obstrução ≥ 50% em vasos com diâmetro ≥ 1,5 mm, seguindo o tutorial do escore SYNTAX^9^ e levando em consideração diversos parâmetros angiográficos.

O escore GRACE foi calculado na admissão dos pacientes, consistindo de oito variáveis: cinco delas computadas de forma semiquantitativa, ou seja, diferente peso para cada estrato de idade, pressão arterial sistólica, frequência cardíaca, creatinina plasmática e classe de Killip; três delas são computadas de forma dicotômica, sendo o infradesnível do segmento ST, elevação de marcador de necrose miocárdica e parada cardíaca na admissão.^10^

### Desfechos Clínicos Hospitalares

Os escores foram testados em relação à predição de dois tipos de desfechos hospitalares, com diferentes conotações: (1) desfechos coronários recorrentes não fatais (infarto, reinfarto ou angina refratária), que representam a complexidade do processo de instabilização coronária; (2) óbito cardiovascular, que representa a incapacidade do organismo de se adaptar ao evento miocárdico isquêmico.

Registrou-se infarto não fatal como a elevação consistente de troponina T ou I, acima dos limites previamente descritos, em pacientes cujos valores estavam negativos nas primeiras 24 horas. Para pacientes com infarto na admissão, um novo pico de CK-MB (≥ 50% do valor prévio e acima do valor normal) foi necessário para a definição de um reinfarto. Elevação de marcadores de necrose relacionados ao procedimento percutâneo ou cirurgia de revascularização não foram registrados como um novo evento. Definiu-se angina refratária como dor precordial recorrente, com pelo menos dois episódios, a despeito do uso de nitrato e controle do duplo produto. Definiu-se morte cardiovascular como morte súbita ou internamento cardiovascular seguido de morte.

### Análise dos Dados

Variáveis categóricas foram expressas em porcentagem. Variáveis numéricas foram expressas em média e desvio-padrão ou mediana e intervalo interquartil nos casos de fuga importante à distribuição normal. Analisou-se a normalidade das variáveis através do teste estatístico de Kolmogorov-Smirnov. Variáveis numéricas foram comparadas com teste *t* de Student não pareado ou Mann-Whitney e categóricas com teste do qui-quadrado ou exato de Fisher.

Foram construídas curvas *Receiver Operating Characteristic* (ROC) dos valores dos escores GRACE e SYNTAX para predição dos desfechos de eventos recorrentes não fatais e óbito cardiovascular, sendo as áreas abaixo da curva (estatística-C) comparadas pelo teste de Hanley-McNeil. Significância estatística foi definida por p < 0,05. SPSS Statistical Software (versão 21.0, SPSS Inc., Chicago, Illinois, EUA) e MedCalc Software (versão 12.3.0.0, Mariakerke, Bélgica) foram utilizados para análise dos dados.

### Cálculo do Tamanho Amostral

A amostra foi dimensionada para oferecer poder estatístico para a comparação das estatísticas-C do SYNTAX versus GRACE: para obter um poder estatístico de 80% (alfa uni-caudal de 0,05) na detecção de 0,05 de superioridade da estatística-C (por exemplo, 0,65 versus 0,70), seria necessário incluir 192 pacientes na análise.

## Resultados

### Descrição da Amostra

Durante o período do estudo, foram incluídos no registro 822 pacientes, sendo que 370 foram submetidos a procedimento de coronariografia, sendo 5 excluídos pois possuíam cirurgia de revascularização prévia. Dos 365 pacientes analisados, a média de idade foi de 64 ± 14 anos, com 58% indivíduos do sexo masculino, sendo 19% portadores de infarto do miocárdio com supradesnivelamento de segmento ST. Doença coronariana com comprometimento triarterial ou de tronco de coronária esquerda esteve presente em 36% da amostra.

A mediana do escore SYNTAX foi de 9 (IIQ = 2,5–20) e do GRACE foi de 117 (IIQ = 90–144). Ao analisarmos os tercis de risco previstos no escore SYNTAX,^11^ 81% dos pacientes apresentou valor baixo (0 a 22), 10% demonstrou valor intermediário (23 a 32) e apenas 8,5% apresentou valor elevado (≥ 33). Em relação ao escore GRACE,^10^ 44% apresentaram risco baixo (< 109), 28% risco intermediário (110 a 139) e 29% alto risco (≥ 140).

A incidência de óbito cardiovascular durante a internação foi 4,4% (16 pacientes) e de eventos isquêmicos não fatais foi 10,7% (39 pacientes). Demais características clínicas estão descritas na Tabela 1 .

**Tabela 1:** Características clínicas e desfechos na amostra

Características clínicas	
Tamanho amostral	365
Idade (anos)	64 ± 14
Sexo masculino	210 (58%)
Isquemia no eletrocardiograma	166 (46%)
Apresentação clínica Angina instável	98 (27%)
Infarto sem supradesnível do ST	196 (54%)
Infarto com supradesnível do ST	71 (19%)
Troponina positiva	232 (64%)
Triarterial ou tronco de coronária esquerda	131 (36%)
Creatinina sérica (mg/dL)	1,0 ± 0,7
Fração de ejeção < 45%	45 (13%)
Classe de Killip > 1	51 (14%)
Escore GRACE *	117 (90–140)
Escore SYNTAX *	9 (2,5–20)
Óbito cardiovascular	16 (4,4%)
Eventos recorrentes não fatais	39 (11%)

IAMSSST: Infarto Agudo do Miocárdio Sem Supradesnível do Segmento ST; IAMCSST: Infarto Agudo do Miocárdio Com Supradesnível do Segmento ST; *: variável exposta como mediana e intervalo interquartil.

### Predição de Desfechos por Escore

**Figura 1:**
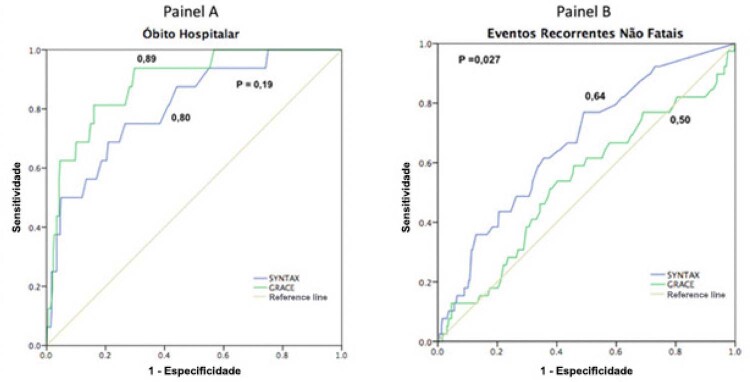
– *Estatística-C da predição de óbito cardiovascular e eventos recorrentes não fatais, evidenciando a acurácia de cada escore em relação ao tipo de desfecho.*

**Figura 2:**
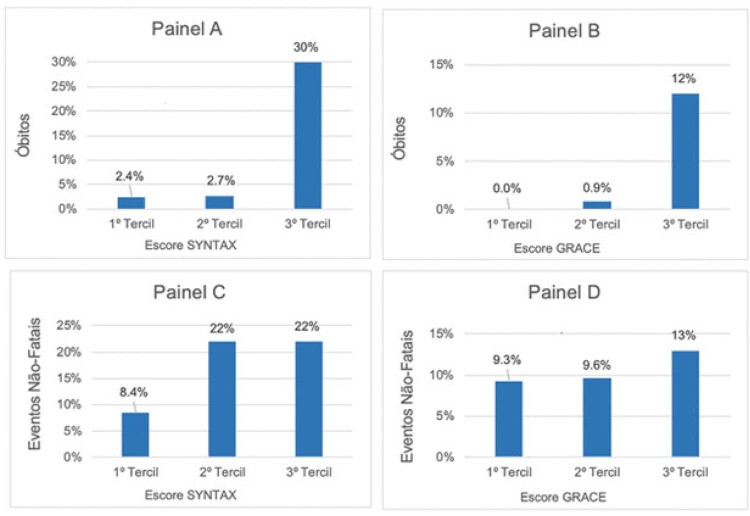
– *Distribuição de desfechos por tercis dos escores SYNTAX e GRACE. Apresentado valor de p < 0,001 no Painel A; p < 0,001 no Painel B; p = 0,007 no Painel C; e p = 0,565 no Painel D.*

Para o desfecho óbito cardiovascular, ambos os escores — SYNTAX e GRACE — apresentaram capacidade discriminatória, com estatísticas-C similares: 0,80 (95%IC: 0,70–0,92) e 0,89 (95%IC: 0,81–0,96), respectivamente — p=0,19 — Figura 1A . Quando os escores foram divididos em tercis de risco, ambos os escores apresentaram aumento de mortalidade no terceiro tercil: respectivamente, 2,4%, 2,7% e 30% no SYNTAX (p < 0,001) e 0%, 0,9% e 12% no GRACE (p < 0,001) – Figura 2 , painéis A e B.

Em relação aos eventos recorrentes não fatais, o escore SYNTAX apresentou valor preditor (estatística-C = 0,64; 95%IC: 0,55–0,73), porém o escore GRACE não mostrou associação com esse tipo de desfecho (estatística-C = 0,50; 95%IC: 0,40–0,61) — p=0,027 — Figura 1B . Quando os escores foram divididos em tercis de risco, o SYNTAX apresentou aumento de desfechos nos dois tercis superiores (8,4%, 22% e 22%, respectivamente, p = 0,007), porém o GRACE não apresentou variação (9,6%, 9,3% e 13%, respectivamente, p = 0,57) – Figura 2 , painéis C e D.

### Características Clínicas Versus Tipo de Desfecho

Pacientes que evoluíram para óbito apresentaram tendência a características clínicas de maior risco, comparados aos sobreviventes. Houve diferença significativa entre os dois grupos quanto a creatinina (3,24 ± 2,6 e 1,15 ± 0,6; p < 0,001), sinais de insuficiência ventricular esquerda aguda (58% e 12%, p < 0,001) e troponina positiva (100% e 72%, p = 0,007), com tendência a diferença em idade, eletrocardiograma isquêmico e pressão arterial – Tabela 2 . Por outro lado, não houve qualquer diferença dessas características entre pacientes que evoluíram com evento não fatal versus pacientes livres de evento – Tabela 3 .

**Tabela 2:** Comparação das características clínicas entre pacientes que apresentaram morte ou sobreviveram ao evento

Variáveis	Óbito	Sobrevida	Valor de p
Tamanho amostral	19	346	
Idade	78 ± 10	63 ± 13	< 0,001
Pressão arterial sistólica	139 ± 32	153 ± 28	0,06
Frequência cardíaca	89 ± 20	79 ± 18	0,03
Creatinina	3,24 ± 2,6	1,15 ± 0,6	<0,001
Killip > 1	9 (48%)	42 (12%)	<0,001
Troponina positiva	19 (100%)	248 (72%)	0,007
ECG isquêmico	10 (53%)	116 (34%)	0,08

**Tabela 3:** Comparação das características clínicas entre pacientes que apresentaram desfecho não fatal versus os livres de desfecho

	Desfecho	Sem desfecho	Valor de p
Tamanho amostral	39	346	
Idade	68 ± 13	64 ± 13	0,05
Pressão arterial sistólica	159 ± 30	152 ± 28	0,15
Frequência cardíaca	74 ± 19	80 ± 18	0,06
Creatinina	0,9 ± 0,3	1,0 ± 0,7	0,058
Killip > 1	5 (13%)	46 (14%)	0,82
Troponina positiva	30 (77%)	237 (73%)	0,58
ECG isquêmico	16 (41%)	110 (34%)	0,36

Ao avaliarmos o evento óbito, a maioria daqueles que apresentaram o desfecho possuíam doença obstrutiva triarterial e/ou comprometimento de tronco de coronária esquerda (81%). Nos sobreviventes, apenas 25% tinha doença triarterial ou de tronco, seguidos de 22% com obstrução de dois vasos, 29% com obstrução de um vaso e 24% livres de lesão obstrutiva (p = 0,01). Naqueles que apresentaram desfechos não fatais, a proporção de livres de coronariopatia obstrutiva, obstrução de um vaso, dois vasos, triarterial/tronco de coronária esquerda foram de 7,7%, 23%, 26% e 44%, respectivamente, comparados a 25%, 29%, 21% e 25%, respectivamente, nos indivíduos livres de eventos (p = 0,04).

## Discussão

O presente estudo propõe um maior refinamento na predição de risco em portadores de síndrome coronariana aguda (SCA). Demonstrou-se que tanto o paradigma clínico (GRACE) como o anatômico (SYTNAX) apresentaram boa capacidade preditora para óbito. Porém, apenas o modelo anatômico foi capaz de predizer eventos recorrentes não fatais. Essa demonstração de que os escores habitualmente utilizados no manejo clínico de portadores de SCA podem possuir uma predileção por diferentes desfechos, até o momento, não havia sido descrita na literatura.

Sabe-se que extensão anatômica da doença coronária é um preditor independente de progressão de placa e eventos coronarianos recorrentes.^12^ Ao avaliarmos o mesmo modelo preditor angiográfico utilizado nesta coorte, estudo prévio com tomografia de coerência ótica demonstrou maior frequência de características compatíveis com vulnerabilidade de placa (placa rica em conteúdo lipídico, fibroateroma de capa fina, ruptura de placa na lesão culpada e múltiplas placas rotas no vaso culpado) em pacientes portadores de SCA com escore SYNTAX mais elevado (≥ 16), do que em tercis do escore baixo (< 9) e intermediário (entre 9 e 16).^13^

Outro estudo realizado em portadores de SCA demonstrou que o escore SYNTAX é um preditor independente de recorrência de infarto, com o melhor valor de SYNTAX de 11 para predição desse desfecho nessa população.^14^ Além disso, o mesmo grupo demonstrou que quanto maior o valor de SYNTAX após a intervenção percutânea, denominada SYNTAX residual, maior a ocorrência de desfechos fatais e não fatais em 30 dias e 1 ano, com valores preditivos e acurácia discriminatória semelhantes ao escore SYNTAX basal (pré-tratamento).^15^ Nosso estudo demonstrou que o escore SYNTAX é um razoável preditor de eventos recorrentes não fatais, estando em consonância com as evidências que associam a carga de doença aterosclerótica a esse tipo de desfecho.

O escore GRACE é um modelo extensamente estudado na predição de eventos cardiovasculares maiores em diversos cenários de SCA.^16^ Apesar disso, há uma paucidade de dados na literatura que avaliem a acurácia preditora desse escore para desfechos não fatais de maneira isolada. A maioria dos trabalhos está associada à predição de eventos combinados ( *Major adverse cardiac events ou, na sigla em inglês* , MACE). As variáveis clínicas presentes nesse modelo refletem o grau de vulnerabilidade do paciente frente ao insulto apresentado em uma SCA e, apesar de predizer complexidade anatômica, esse modelo não possui uma boa acurácia preditora, conforme dados previamente demonstrados por nosso grupo.^19 - 21^ O trabalho atual não foi capaz de demonstrar associação entre o escore GRACE e a ocorrência de novos eventos isquêmicos não fatais.

Do ponto de vista mecanicista, a diferença entre os achados dos referidos escores pode ser interpretada através das características das variáveis que cada um analisa. O escore GRACE utiliza em sua composição variáveis relacionadas ao aspecto clínico do paciente e, de certo modo, se associa ao potencial risco de instabilidade de uma ampla gama de pacientes. No entanto, não se correlaciona diretamente com a instabilidade coronariana, uma vez que, pela sua composição, não é possível identificar com propriedade a gravidade das lesões existentes. Por outro lado, o escore SYNTAX, utilizado como o paradigma anatômico, se baseia justamente na gravidade das lesões coronarianas existentes e consegue preencher a lacuna deixada pelo escore anterior.

Ademais, novos eventos coronarianos (recorrência de SCA) potencialmente influenciam a predição de mortalidade, pois infarto causa morte. Por outro lado, óbito cardiovascular como evento inicial não teria como influenciar a ocorrência de evento recorrente subsequente. Essa óbvia observação reforça a lógica dos nossos resultados de que quando eventos recorrentes são preditos, morte também é (escore SYNTAX); porém; a predição de morte decorrente de um insulto cardíaco não garante predição de eventos isquêmicos recorrentes (escore GRACE).Trata-se de um estudo gerador de hipótese, que evidenciou a eventual necessidade de discriminar os desfechos decorrentes de uma SCA, definindo uma utilidade prática para os modelos preditores clínico e anatômico. A utilização de desfechos combinados surgiu nos grandes registros e ensaios clínicos para resolver potenciais limitações de poder estatístico. No entanto, esse método institui um mesmo peso para desfechos diversos, não distinguindo a significância relativa de cada um.^22^ As implicações práticas deste estudo residem na necessidade de avaliarmos, dentro do contexto clínico-anatômico, a probabilidade isolada dos diferentes desfechos, além de reconhecermos a limitação do conhecimento de dados clínicos em predizer eventos coronarianos recorrentes. Isso poderia influenciar o processo de tomada de decisão para o tratamento de portadores de SCA, onde o risco clínico inicial habitualmente dita a magnitude do tratamento. Este estudo refuta essa prática, pois diante de um indivíduo com GRACE baixo, ainda haveria a possibilidade de haver um risco angiográfico elevado. Sendo assim, uma predição de eventos global, levando-se em consideração modelos preditores complementares e a predileção por desfechos diversos o melhor caminho para uma adequada estratificação de risco. Este estudo possui como principal limitação o seu tamanho amostral, podendo estar sujeito a erro tipo II. Além disso, apesar de utilizarmos dois escores frequentemente utilizados na prática clínica, ainda assim seria interessante uma avaliação comparativa dos demais escores clínicos e anatômicos para a predição de diferentes desfechos, sob o prisma dos paradigmas anatômico e clínico.

## Conclusão

Em conclusão, o presente estudo indica que dados anatômicos contribuem para a predição de eventos recorrentes não fatais e óbito cardiovascular em SCA. Por outro lado, dados clínicos são capazes de predizer morte, mas não influenciam a probabilidade de desfechos não fatais.
