# Supplementary material for: Competência Prognóstica Distinta entre Modelo Clínico e Anatômico em Síndromes Coronarianas Agudas: Comparação por Tipo de Desfecho
Source: Arq Bras Cardiol. 2020 Aug 19;115(2):219–25. [Article in Portuguese] doi: 10.36660/abc.20190062 (PMC8384280; doi:10.36660/abc.20190062)
Supplement: Translation

# Translation: Prognostic Contrast between Anatomical and Clinical Models Regarding Fatal and Non-Fatal Outcomes in Acute Coronary Syndromes

**Authors:** Mateus S. Viana, Vitor C. A. Correia, Felipe M. Ferreira, Yasmin F. Lacerda, Gabriela O. Bagano, Leticia L. Fonseca, Lara Q. Kertzman, Milton V. Melo, Marcia M. Noya-Rabelo, Luis C L Correia

**Affiliations:** 1 Brazil Escola Bahiana de Medicina e Saúde Pública, Salvador, BA - Brazil; 2 Brazil Hospital São Rafael, Salvador, BA – Brazil

**Keywords:** Acute Coronary Syndrome/physiopathology, Atherosclerosis, Myocardial Infarction, Mortality, Cardiovascular Diseases/prevention and control, Hospitalization, Prognosis

## Abstract

**Background:**

Recurrent ischemic events are mediated by atherosclerotic plaque instability, whereas death after an ischemic event results from gravity of insult and ability of the organism to adapt. The distinct nature of those types of events may respond for different prediction properties of clinical and anatomical information regarding type of outcome.

**Objective:**

To identify prognostic properties of clinical and anatomical data in respect of fatal and non-fatal outcomes of patients hospitalized with acute coronary syndromes (ACS).

**Methods:**

Patients consecutively admitted with ACS who underwent coronary angiography were recruited. The SYNTAX score was utilized as an anatomic model and the GRACE score as a clinical model. The predictive capacity of those scores was separately evaluated for prediction of non-fatal ischemic outcomes (infarction and refractory angina) and cardiovascular death during hospitalization. It was considered as significant a p-value <0,05.

**Results:**

EAmong 365 people, cardiovascular death was observed in 4,4% and incidence of non-fatal ischemic outcomes in 11%. For cardiovascular death, SYNTAX and GRACE score presented similar C-statistic of 0,80 (95% IC: 0,70 – 0,92) and 0,89 (95% IC 0,81 – 0,96), respectively – p = 0,19. As for non-fatal ischemic outcomes, the SYNTAX score presented a moderate predictive value (C-statistic = 0,64; 95%IC 0,55 – 0,73), whereas the GRACE score did not presented association with this type of outcome (C-statistic = 0,50; 95%IC 0,40-0,61) – p = 0,027.

**Conclusion:**

Clinical and anatomic models similarly predict cardiovascular death in ACS. However, recurrence of coronary instability is better predicted by anatomic variables than clinical data. (Arq Bras Cardiol. 2020; [online].ahead print, PP.0-0)

## Introduction

Multivariate models have been validated as prognostic tools in acute coronary syndromes (ACS), consisting of clinical data,^1^ anatomical data^2^ or a combination of the two.^3 - 6^ These models have recognized predictive value for recurrent events, but it is not clear whether the prognostic value varies depending on the type of outcome.

Recurrent non-fatal ischemic events represent the phenomenon of atherosclerotic plaque instability, while death after an ischemic event results from the severity of the insult and the resistance of the organism. The different pathophysiological nature of these types of events can cause clinical and anatomical data to have different prognostic capacities depending on the type of outcome. If this is true, the generalization of the prognostic value regarding “cardiovascular outcomes” would be compromised, making it necessary to individualize the prediction of each model for the type of outcome.

This work aims to evaluate and compare the prognostic value of clinical and anatomical data in relation to fatal and non-fatal outcomes in patients with ACS. Thus, a hospital cohort with patients admitted under these conditions was performed, the GRACE score chosen as the representative of the prediction for clinical data and the SYNTAX score used as a predictor based on anatomy.

## Metodology

### Study Population

Individuals consecutively admitted to the Intensive Cardiovascular Unit of a tertiary hospital between July 2007 and September 2014, with a diagnosis of ACS, were selected. The inclusion criterion was defined by typical or equivalent precordial discomfort and at rest in the last 48 hours, associated with at least one of the following characteristics: 1) positive myocardial necrosis marker, defined by troponin T ≥ 0.01 μg / L or troponin I ≥ 0.034 μg / L, which corresponds to values above the 99th percentile;^7 , 8^ 2) ischemic electrocardiographic changes, consisting of T wave inversion (≥ 0.1 mV) or ST segment deviation (≥ 0, 05 mV); 3) documented coronary artery disease, defined by a history of myocardial infarction or previous angiography showing coronary obstruction ≥ 50% of the luminal diameter. In addition, for inclusion in the analysis, patients needed to have undergone a coronary angiography procedure during hospitalization. Those who disagreed with participating in the registry and / or who had previously undergone surgical myocardial revascularization procedures were excluded. The protocol is in accordance with the Declaration of Helsinki, and was released by the Institutional Research Ethics Committees. All patients signed an informed consent form.

### Predicative Scores (SYNTAX e GRACE)

The SYNTAX score was calculated by an experienced interventional cardiologist who was blind to the clinical picture and the outcomes. This physician assessed each obstruction of the coronary tree with an obstruction percentage ≥ 50% in vessels with a diameter ≥ 1.5 mm, following the SYNTAX score tutorial^9^ and considering various angiographic parameters.

The GRACE score was calculated on patient admission, consisting of eight variables: five of them computed in a semi-quantitative way, that is, different weight for each age group, systolic blood pressure, heart rate, plasma creatinine and Killip class; three of them are computed in a dichotomous way, with the ST segment elevation, elevation of myocardial necrosis marker and cardiac arrest at admission.^10^

### Hospital Clinical Outcomes

The scores were tested in relation to the prediction of two types of hospital outcomes with different connotations: (1) non-fatal recurrent coronary outcomes (infarction, re-infarction or refractory angina), which represent the complexity of the coronary instability process; (2) cardiovascular death, which represents the body’s inability to adapt to the ischemic myocardial event.

Nonfatal infarction was recorded as a consistent elevation of troponin T or I, above the previously described limits, in patients whose values were negative in the first 24 hours. For patients with infarction on admission, a new peak of CK-MB (≥ 50% of the previous value and above the normal value) was necessary to define a re-infarction. Elevation of necrosis markers related to percutaneous procedure or revascularization surgery were not recorded as a new event. Refractory angina was defined as recurrent chest pain, with at least two episodes, despite the use of nitrate and double product control. Cardiovascular death was defined as sudden death or cardiovascular hospitalization followed by death.

### Data Analysis

Categorical variables were expressed as a percentage. Numerical variables were expressed as mean and standard deviation or median and interquartile range in cases of significant leak to normal distribution. The normality of the variables was analyzed using the Kolmogorov-Smirnov statistical test. Numerical variables were compared with the unpaired Student’s t-test or Mann-Whitney and categorical with the chi-square test or Fisher’s exact test.

Receiver Operating Characteristic (ROC) curves were constructed for the GRACE and SYNTAX score values to predict the outcomes of non-fatal recurrent events and cardiovascular death, with the areas below the curve (C-statistic) compared by the Hanley-McNeil test. Statistical significance was defined by p <0.05. SPSS Statistical Software (version 21.0, SPSS Inc., Chicago, Illinois, USA) and MedCalc Software (version 12.3.0.0, Mariakerke, Belgium) were used for data analysis.

### Sample Size Calculation

The sample was sized to offer statistical power for the comparison of SYNTAX C-statistics versus GRACE: to obtain 80% statistical power (one-tailed alpha of 0.05) in detecting 0.05 superiority of C-statistics (for example, 0.65 versus 0.70), it would be necessary to include 192 patients in the analysis.

## Results

### Sample Description

During the study, 822 patients were included in the register, wherein 370 were submitted to coronarography procedure, while 05 were excluded once they already have previous revascularization surgery. Of the 365 patients analyzed, the media of age was 64 ± 14 years old, 58% were male, 19% of whom had ST-segment elevation myocardial infarction. Coronary Disease with tri-arterial or left main coronary involvement was present in 36% of the sample.

The median of SYNTAX Score was 9 (IQR = 2,5 - 20) and GRACE’s 117 (IQR = 90 -144). Analyzing the risk terciles predicted in the SYNTAX Score,^11^ 81% of patients had a low value (0 to 22), 10% had an intermediate value (23 to 32) and only 8.5% had high value (≥ 33). Regarding the GRACE score,^10^ 44% had low-risk (<109), 28% had intermediate-risk (110 to 139) and 29% had high-risk (≥ 140).

The incidence of cardiovascular death during hospitalization was 4.4% (16 patients) and non-fatal ischemic events were 10.7% (39 patients). Other clinical characteristics are described in Table 1 .

**Table 1:** Clinical Characteristics and sample outcomes

Clinical Characteristics	
Sample Size	365
Age (years)	64 ± 14
Male	210 (58%)
Ischemia signs in electrocardiogram	166 (46%)
Clinical presentation	
Unstable Angina	98 (27%)
Non-ST-elevation myocardial infarction	196 (54%)
ST-elevation myocardial infarction	71 (19%)
Positive troponin	232 (64%)
Tri-arterial or left main coronary	131 (36%)
Serum creatine (mg/dl)	1,0 ± 0,7
Ejection fraction < 45%	45 (13%)
Killip class > 1	51 (14%)
GRACE Score*	117 (90 – 140)
SYNTAX Score*	9 (2,5 – 20)
Cardiovascular death	16 (4,4%)
Non-fatal recurrent events	39 (11%)

NSTEMI Non-ST-elevation myocardial infarction; STEMI: ST-elevation myocardial infarction; *: variable exposed as median and interquartile range.

### Outcome Predictions by Score

For death outcome, SYNTAX and GRACE scores presented discriminatory capacity, with similar c-statistic: 0,80 (95% CI: 0,70 – 0,92) and 0,89 (95% CI 0,81 – 0,96), respectively — p = 0,019 — Figure 1A . When the scores were divided into risk terciles, both scores showed an increase of mortality in the third tercile: respectively, 2,4%, 2,7% and 30% on SYNTAX (P < 0,001) and 0%, 0,9% and 12% on GRACE (p < 0,001) — Figure 2 , panel A and B.

**Figure 1:** – *C-statistic of cardiovascular death prediction and non-fatal recurrent events, evidencing the accuracy of each score in relation to the type of outcome.*

**Figure 2:** – *Outcome distribution by terciles of the SYNTAX and GRACE scores. A value of p <0.001 was shown in Panel A; p < 0,001 on Panel B; p = 0,007 on Panel C; and p = 0,565 on Panel D.*

Regarding non-fatal recurrent events, SYNTAX score presented a predictor value (C-statistic = 0,64; 95% CI 0,55 -0,73). However, the GRACE score showed no association with this type of outcome (C-statistic = 0,50; 95% CI 0,40 – 0,61) — p=0,027 — Figure 1B . When the scores were divided into risk terciles, SYNTAX showed an increase in outcomes in the upper two terciles (8,4%, 22% and 22%, respectively, p = 0,007). However, GRACE showed no variation (9,6%, 9,3% and 13%, respectively, p = 0,57) — Figure 2 , panels C e D.

### Clinical Characteristics versus Type of outcome

Patients who evolved to death showed a tendency to higher risk clinical characteristics, compared to survivors. There was a significant difference between the two groups regarding creatinine (3,24 ± 2,6 and 1,15 ± 0,6; p < 0,001), acute left ventricular failure signs (58% and 12%, p < 0,001) and positive troponin (100% and 72%, p = 0,007), with a trend in age difference, ischemic electrocardiogram and blood pressure — Table 2 . Nevertheless, there wasn’t any difference of those characteristics between patients who evolved with a non-fatal event versus event-free patients — Table 3 .

**Table 2:** Comparing Clinical Characteristics between patients who died versus those who survived the event

Variables	Death	Survival	p value
Sample Size	19	346	
Age	78 ± 10	63 ± 13	< 0,001
Systolic Arterial Hypertension	139 ± 32	153 ± 28	0,06
Heart rate	89 ± 20	79 ± 18	0,03
Creatine	3,24 ± 2,6	1,15 ± 0,6	<0,001
Killip > 1	9 (48%)	42 (12%)	<0,001
Positive Troponin	19 (100%)	248 (72%)	0,007
Ischemic ECG	10 (53%)	116 (34%)	0,08

**Table 3:** Comparison of clinical characteristics between patients who presented non-fatal outcome Versus those free of outcomes

	Outcome	No Outcome	p Value
Sample Size	39	346	
Age	68 ± 13	64 ± 13	0.05
Systolic Arterial	159 ± 30	152 ± 28	0.15
Hypertension			
Heart Rate	74 ± 19	80 ± 18	0.06
Creatine	0.9 ± 0.3	1.0 ± 0.7	0.058
Killip > 1	5 (13%)	46 (14%)	0.82
Positive Troponin	30 (77%)	237 (73%)	0.58
Ischemic ECG	16 (41%)	110 (34%)	0.36

When evaluating the death event, most of those who showed this outcome already had a tri-arterial obstructive disease and/or left main coronary involvement (81%). In the survivors, only 25% had tri-arterial or left main coronary disease, followed by 22% with obstruction of two vessels, 29% with obstruction of one vessel and 24% free of obstructive injury (P ≤ 0,01). On those who showed non-fatal outcomes, the proportion of non-obstructive coronaropathy, one vessel obstruction, two vessels obstruction, tri-arterial/left main coronary were 7,7%, 23%, 26% e 44%, respectively, comparing to 25%, 29%, 21% and 25%, respectively, in free-event individuals (P=0,04)

## Discussion

The present study requested a further refinement in risk prediction in patients with acute coronary syndrome (ACS). It was demonstrated how well the clinical paradigm (GRACE) as anatomical (SYTNAX) showed good capacity attributed to death, however only the anatomical model was able to predict recurrent non-fatal events. This demonstration that the scores commonly used in the clinical laboratory of patients with ACS may use a predilection for different outcomes, so far, have not been used in the literature.

It is known that anatomical extension of coronary disease is an independent predictor of plaque progression and recurrent coronary events.^12^ When evaluating the same angiographic predictor model used in this cohort, a previous study with optical coherence tomography demonstrated a higher frequency of characteristics compatible with vulnerability of plaque (plaque rich in lipid content, thin-cap fibroatheroma, rupture of plaque in the culprit lesion and multiple broken plaques in the culprit vessel) in patients with ACS with a higher SYNTAX score (≥16) than in tertiles of the low score (<9) and intermediate (between 9 and 16).^13^

Another study carried out in patients with ACS demonstrated that the SYNTAX score is an independent predictor of infarction recurrence, with the best SYNTAX value of 11 to predict this outcome in this population.^14^ In addition, the same group demonstrated that the higher the value of SYNTAX after percutaneous intervention, which was called Residual SYNTAX, there was an association with a higher occurrence of fatal and non-fatal outcomes in 30 days and 1 year, with predictive values and discriminatory accuracy similar to the baseline SYNTAX score (pre-treatment).^15^ Our study demonstrated that the SYNTAX score is a reasonable predictor of recurrent non-fatal events, in line with the evidence that associates the burden of atherosclerotic disease with this type of outcome.

The GRACE score is a model extensively studied in the prediction of major cardiovascular events in different ACS scenarios.^16 - 18^ Despite this, there is a paucity of data in the literature that assess the predictive accuracy of this score for non-fatal outcomes in isolation. Most studies are associated with combined event prediction (MACE). The clinical variables present in this model reflect the patient’s degree of vulnerability to the insult presented in an ACS and, despite predicting anatomical complexity, it does not have a good predictive accuracy, according to data previously demonstrated by our group.^19 - 21^ The current study has not been able to demonstrate an association between the GRACE score and the occurrence of new non-fatal ischemic events.

From a mechanistic point of view, the difference between the findings of these scores can be interpreted through the characteristics of the variables that each one analyzes. The GRACE score uses in its composition variables related to the clinical aspect of the patient and, in a way, is associated with the potential risk of instability in a wide range of patients. However, it is not directly correlated with coronary instability, since due to its composition it is not possible to properly identify the severity of existing lesions. On the other hand, the SYNTAX score, used as the anatomical paradigm, is based precisely on the severity of existing coronary lesions and manages to fill the gap left by the previous score. In addition, new coronary events (ACS recurrence) potentially influence the prediction of mortality, since infarction causes death. On the other hand, cardiovascular death as an initial event could not influence the occurrence of a subsequent recurrent event. This obvious observation reinforces the logic of our results that when recurrent events are predicted, death is also (SYNTAX score), but the prediction of death due to a cardiac insult does not guarantee prediction of recurrent ischemic events (GRACE score). It is a hypothesis-generating study, which evidenced an eventual need to discriminate the outcomes resulting from an ACS, defining a practical utility for the clinical and anatomical predictive models. The use of combined outcomes has emerged in large registries and clinical trials to address potential limitations of statistical power, however this method establishes the same weight for different outcomes, not distinguishing the relative significance of each one.^22^ The practical implications of this study lie in the need to assess, within the clinical-anatomical context, the isolated probability of different outcomes, in addition to recognizing the limited knowledge of clinical data in predicting recurrent coronary events. This could influence the decision-making process for the treatment of patients with ACS, where the initial clinical risk usually dictates the magnitude of the treatment. This study refutes this practice, because in the face of an individual with low GRACE, there would still be the possibility of a high angiographic risk. Thus, a global event prediction, taking into account complementary predictor models and a predilection for different outcomes, is the best way for an adequate risk stratification.

The main limitation of this study is its sample size, which may be subject to type II error. In addition, although we use two scores frequently used in clinical practice, it would still be interesting to have a comparative assessment of the other clinical and anatomical scores for the prediction of different outcomes, from the perspective of the anatomical and clinical paradigms.

## Conclusion

In conclusion, the present study indicates that anatomical data contribute to the prediction of recurrent non-fatal events and cardiovascular death in ACS. On the other hand, clinical data are able to predict death, but do not influence the likelihood of non-fatal outcomes.
